# Structural equation modeling for investigating multi-trait genetic architecture of udder health in dairy cattle

**DOI:** 10.1038/s41598-020-64575-3

**Published:** 2020-05-08

**Authors:** Sara Pegolo, Mehdi Momen, Gota Morota, Guilherme J. M. Rosa, Daniel Gianola, Giovanni Bittante, Alessio Cecchinato

**Affiliations:** 1 Department of Agronomy, Food Natural resources, Animals and Environment, University of Padua, Legnaro, (PD) Italy; 2 Department of Animal and Poultry Sciences, Virginia Polytechnic Institute and State University, Blacksburg, VA USA; 3 Department of Animal Sciences, University of Wisconsin, Madison, WI USA; 4 Department of Biostatistics and Medical Informatics, University of Wisconsin, Madison, WI USA; 5 Department of Dairy Science, University of Wisconsin, Madison, WI USA

**Keywords:** Animal breeding, Genome-wide association studies

## Abstract

Mastitis is one of the most prevalent and costly diseases in dairy cattle. It results in changes in milk composition and quality which are indicators of udder inflammation in absence of clinical signs. We applied structural equation modeling (SEM) - GWAS aiming to explore interrelated dependency relationships among phenotypes related to udder health, including milk yield (MY), somatic cell score (SCS), lactose (%, LACT), pH and non-casein N (NCN, % of total milk N), in a cohort of 1,158 Brown Swiss cows. The phenotypic network inferred via the Hill-Climbing algorithm was used to estimate SEM parameters. Integration of multi-trait models-GWAS and SEM-GWAS identified six significant SNPs for SCS, and quantified the contribution of MY and LACT acting as mediator traits to total SNP effects. Functional analyses revealed that overrepresented pathways were often shared among traits and were consistent with biological knowledge (e.g., membrane transport activity for pH and MY or Wnt signaling for SCS and NCN). In summary, SEM-GWAS offered new insights on the relationships among udder health phenotypes and on the path of SNP effects, providing useful information for genetic improvement and management strategies in dairy cattle.

## Introduction

Biological systems are pervaded by causal relationships among variables. Structural equation models (SEM)^1^ can be used to represent causal relationships among phenotypic traits and infer their magnitude^2^. The use of SEM in the context of quantitative genetics was first described by Gianola and Sorensen^3^. SEM delivers an interpretation of results that is different from that of multi-trait models (MTM), which can only capture covariances and correlations among variables and do not consider the existence of recursive and feedback mechanisms^4^. In contrast to MTM, SEM explore functional links between variables in a phenotype network, in which one trait can be considered as a predictor of another trait^5^. In dairy cattle, some attempts have been made to describe the complex relationships and identify possible causal paths among traits related to calving^6^, health and fertility^7^, risk and tolerance to mastitis^8^, and milk composition^9,10^ using the SEM approach.

In genome-wide association studies (GWAS), SEM might offer a powerful and flexible tool to capture causal structures, which are missed through MTM-GWAS and, therefore, to more accurately describe the associations between traits and quantitative trait loci (QTL). In particular, SEM-GWAS is able to decompose single-nucleotide polymorphisms (SNP) effects on a trait into direct or indirect components (i.e., mediated by an up-stream trait in the network) and also to identify genomic regions with pleiotropic effects explaining observed genetic correlations. This methodology has been applied to some economically important characteristics in broiler chickens^11^ and beef cattle^12^, but to our knowledge, no study is available in dairy cattle.

In this study, we hypothesized that possible dependency relationships exist among a set of traits related to udder health. Mastitis is one of the most prevalent production diseases in dairy herds worldwide^13^. In particular, sub-clinical mastitis represents a continuous risk of infection for the whole stock and can cause heavy financial losses and large nutritional and technological impacts^14^. Moreover, animal welfare aspects and the risk of antibiotic residues in dairy products are other important critical points^15^. The prevention or detection at an early stage is important for both animal welfare and human health. Milk somatic cell count (SCC) has been extensively considered as the most effective indicator of mastitis^16^ and it is included in sire genetic evaluation procedures in several countries^17^. However, inflammation occurring after the entrance and multiplication of pathogenic microorganisms in the mammary gland activates a complex series of events leading not only to an elevated SCC but also to a reduced synthetic activity and milk compositional changes^14,18^, which affect milk quality and hygiene and, indirectly, also its technological characteristics. For instance, intra-mammary infection causes enhanced leakage of lactose from milk to blood due to the damage of blood-milk barrier^19^. The increase in tight junction permeability is accompanied by a decrease in the rate of milk synthesis and secretion of the major specific milk constituents^20^. In particular, the concentration of caseins is reduced in infected quarters as a result of the reduced secretion and increased proteolysis^21^. As a consequence, the casein to total protein ratio is negatively affected due to the increase in non-protein N^18^. An increase in the concentrations of proteins of blood serum origin, including serotransferrin and albumin, was also detected in mastitic whey^22^. Changes in ionic equilibrium often due to increased amounts of sodium and chloride and reduced potassium ion concentrations in mastitic milk have been reported to explain the increase in milk pH^23^. Accordingly, the use of synthetic indices of udder health, which include not only SCC but also other indicator traits such as milk lactose and pH has been recently proposed^24,25^. The relationships among these phenotypes and between these phenotypes and udder health are therefore well-known but the existence of possible dependency paths has not been explored.

Therefore, the aims of this study were: (1) to use probabilistic graphical models for investigating the interrelationships among traits related to udder-health, namely milk yield (MY), lactose percentage (LACT), pH, non-casein N (NCN, expressed as percentage of total milk N) and somatic cell score (SCS); and (2) to use the inferred network structure to estimate SEM parameters and carry out SEM-GWAS analysis to partition SNP effects into direct, indirect (i.e. mediated by an up-stream phenotype in the network), and total effects.

## Results

### Phenotypic correlations and network structure

Descriptive statistics for the traits investigated are reported in Table 1. Average values were 24.72 kg/day (±7.62) for MY, 6.64 (±0.08) for milk pH, 4.87% (±0.18) for LACT, and 21.96% (±1.23) for NCN. SCS averaged 2.85 and it has large variability (±1.83). Genomic and residual correlations together with heritability estimates obtained with a multi-trait Bayesian GBLUP model are reported in Table 1. The only statistically relevant estimate of genomic correlation was that obtained between LACT and NCN (-0.280). Among residual correlations, we found relevant positive correlations between LACT and MY (0.142) and between SCS and NCN (0.287), and negative between SCS and LACT (−0.420) and between NCN and LACT (−0.561). Heritability estimates were large (>0.35) for milk pH and LACT, moderate for NCN (0.214), and low for MY (0.130) and SCS (0.107).

**Table 1:** Descriptive statistics, genomic (upper triangular) and residual (lower triangular) correlations, and genomic heritabilities (diagonals) for the milk traits.

	Mean_(SD)_	MY	pH	LACT	SCS	NCN
MY (kg/d)	24.72_(7.62)_	0.130_(0.004;0.245)_	−0.006_(−0.149;0.140)_	−0.063_(−0.330;0.215)_	0.475_(−0.048;0.880)_	0.377_(−0.060;0.795)_
pH	6.64_(0.08)_	−0.001_(−0.092;0.085)_	0.515_(0.462;0.567)_	0.016_(−0.111;0.137)_	0.017_(−0.128;0.159)_	−0.023_(−0.157;0.124)_
LACT(%)	4.87_(0.18)_	**0.142**_**(0.047;0.236)**_	−0.046_(−0.142;0.053)_	0.388_(0.316;0.463)_	−0.190_(−0.417;0.042)_	−**0.280**_**(−0.475;−0.074)**_
SCS	2.85_(1.83)_	−0.076_(−0.171;0.016)_	0.066_(−0.018;0.154)_	**−0.420**_**(−0.495;−0.347)**_	0.107_(0.037;0.182)_	0.271_(−0.679;0.164)_
NCN(%)	21.96_(1.23)_	−0.073_(−0.177;0.030)_	0.020 _(−0.070 ;0.111;)_	-**0.561**_**(−0.630;−0.491)**_	**0.287**_**(0.199;0.371)**_	0.214_(0.110;0.321)_

MY: milk yield; LACT: lactose; SCS:somatic cells score, calculated as log2 (somatic cell count/100,000) + 3; NCN: casein (non-casein N expressed as % of total milk N)

Lower and upper bounds of the highest 95% probability density regions (HPD95) obtained from the estimated marginal densities are given in parantheses. Relevant correlations (HPD95 not including 0) are highlighted in bold.

**Figure 1:**
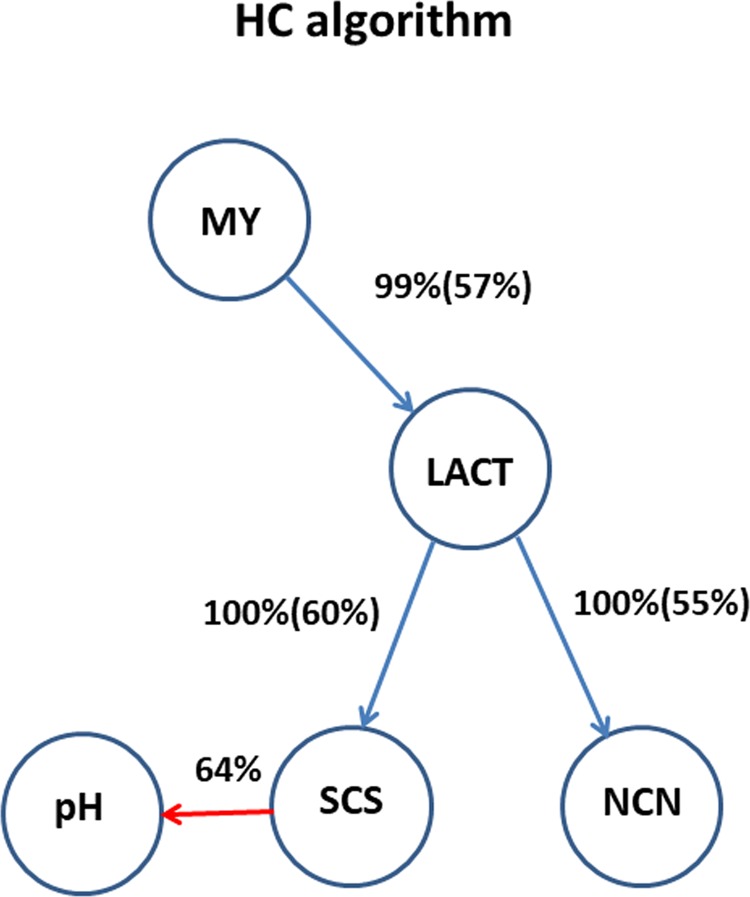
Network structure inferred from the vector of the residuals using the Hill-Climbing (HC) algorithm. Network structure inferred combining the results obtained with HC algorithm and prior biological knowledge (for trait pH). Structure learning test was performed with 50,000 bootstrap samples. The percentages reported beside edges indicate the proportion of bootstrap samples supporting the edge and (in parentheses) the proportion having the direction shown.

Bayesian network structure learning algorithms were applied to the vector of residuals from the Bayesian GBLUP analysis to identify putative dependencies among phenotypes free of “genomic confounders”. The results obtained with the HC algorithm are displayed in Fig. 1. In this network, we found a direct dependence between MY and LACT (57% of bootstrap samples). The paths between MY and SCS and between MY and NCN were mediated by LACT. Direct connections were also found between LACT and SCS (60% of bootstrap samples) and between LACT and NCN (55% of bootstrap samples). The algorithm was not able to detect a relationship between pH and the other traits with high confidence (>85% edge strength). Therefore, we integrated statistical inference with prior biological information to set an arc with direction SCS → pH (Fig. 1). The largest decrease in BIC was observed when removing the arcs LACT → NCN and LACT → SCS, which suggested that these paths might play the most important roles in the network (Table 2).

**Table 2:** Bayesian Information Criterion (BIC) score for the Hill-Climbing (HC) algorithm and path coefficients derived from the structural equation models.

BIC^a^	Path	BIC^b^	λ	Path coefficient
−3490.46	MY → LACT	−14.71	λ_21_	−0.065
	LACT → SCS	−133.87	λ_32_	−0.632
	SCS → pH	−1.49	λ_43_	0.057
	LACT → NCN	−261.55	λ_52_	−0.262

^a^Bayesian information criterion score (BIC) for the entire network.

^b^BIC scores for pairs of nodes; the change in the score when removing the arc relative to the entire network score is showed.

MY: milk yield; LACT: lactose percentage; pH: milk pH; SCS: somatic cell score; NCN: non-casein N(expressed as % of total milk N).

### Structural equation coefficients

**Figure 2:**
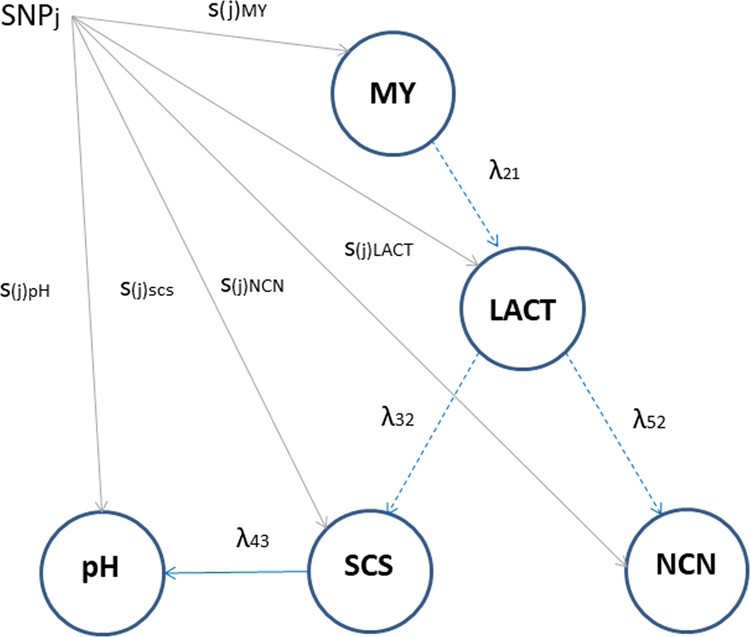
A scheme for path analysis of SNP effects for five milk-related traits. MY: milk yield; pH: milk pH; LACT: lactose; SCS: somatic cell score; NCN: casein (expressed as % of total milk N). The blue arrows indicate the direction of relationship according to the learned causal structure. Dashed lines correspond to a negative path coefficient. λ_21_: MY → LACT; λ_32_: LACT → SCS; λ_43_: SCS → pH; λ_52_: LACT → NCN (non-casein N, expressed as % of total milk N). The grey arrows correspond to the direct effect of SNP_*j*_ on the trait.

We modelled the inferred Bayesian network for MY, LACT, SCS, pH, and NCN (Fig. 1) with a set of SEM equations reported in Supplementary material S1 from which parameters and SNP effects were estimated. The corresponding directed acyclic graph is shown in Fig. 2, which represents all the recursive relationships among the five phenotypes. The estimated path coefficients are reported in Table 2. The paths MY → LACT, LACT → SCS and LACT → NCN had negative coefficients, while SCS → pH had a positive coefficient. The path LACT → SCS had the largest structural coefficient (−0.632) while SCS → pH had the lowest one (0.057).

### Partitioning of SNP effects

**Figure 3:**
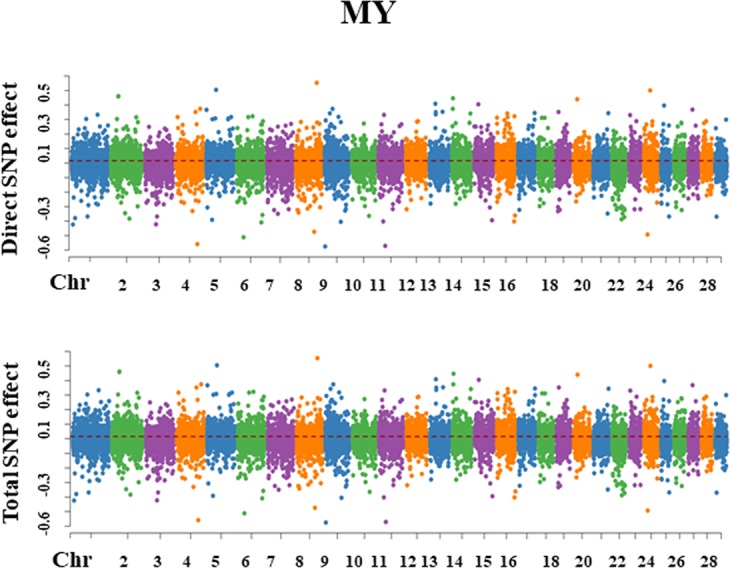
Manhattan plots for SNP effects on milk yield obtained using SEM-GWAS based on the network structure learned by Hill-Climbing algorithm. MY: milk yield.

**Figure 4:**
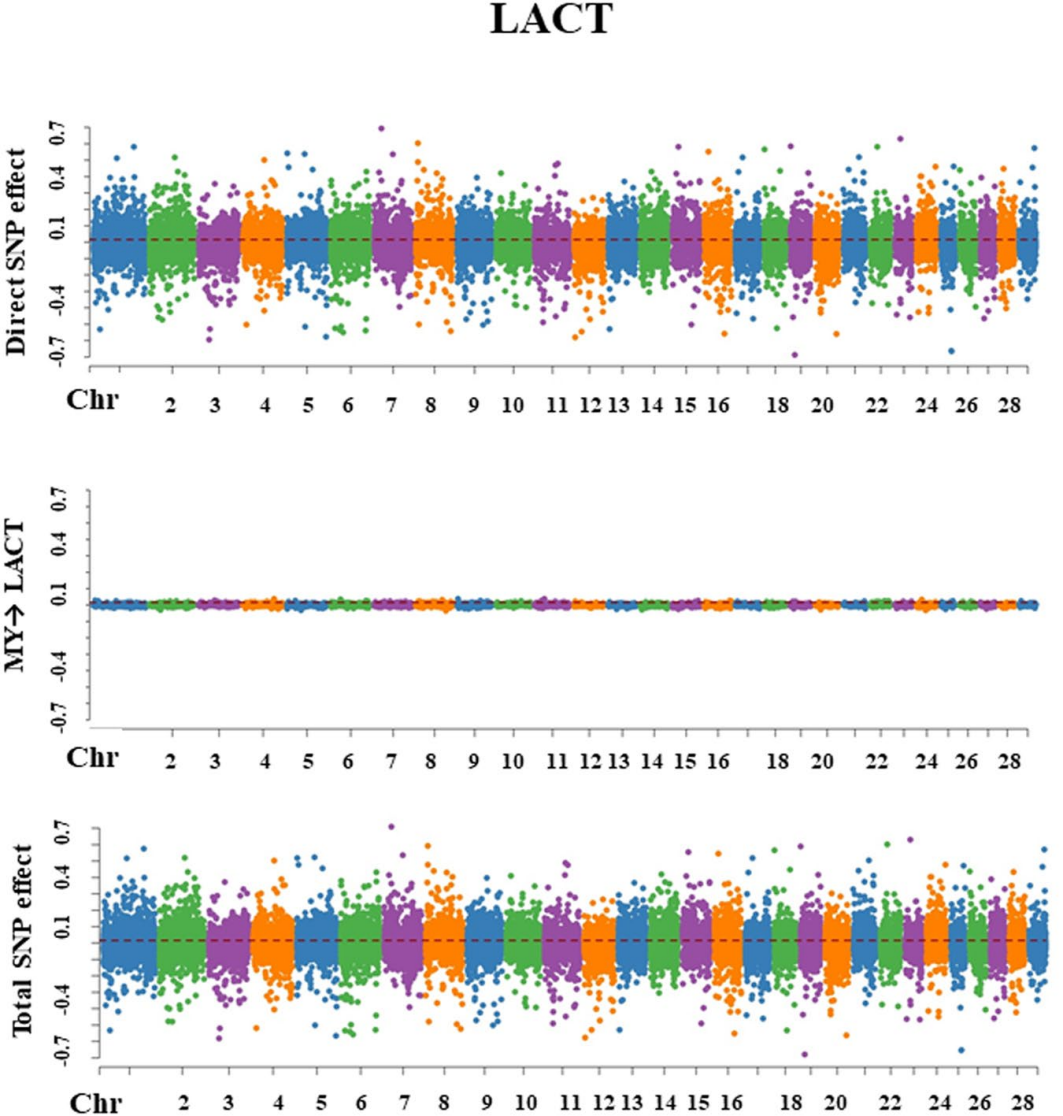
Manhattan plots for SNP effects on milk lactose obtained using SEM-GWAS based on the network structure learned by Hill-Climbing algorithm. LACT: lactose; MY: milk yield.

**Figure 5:**
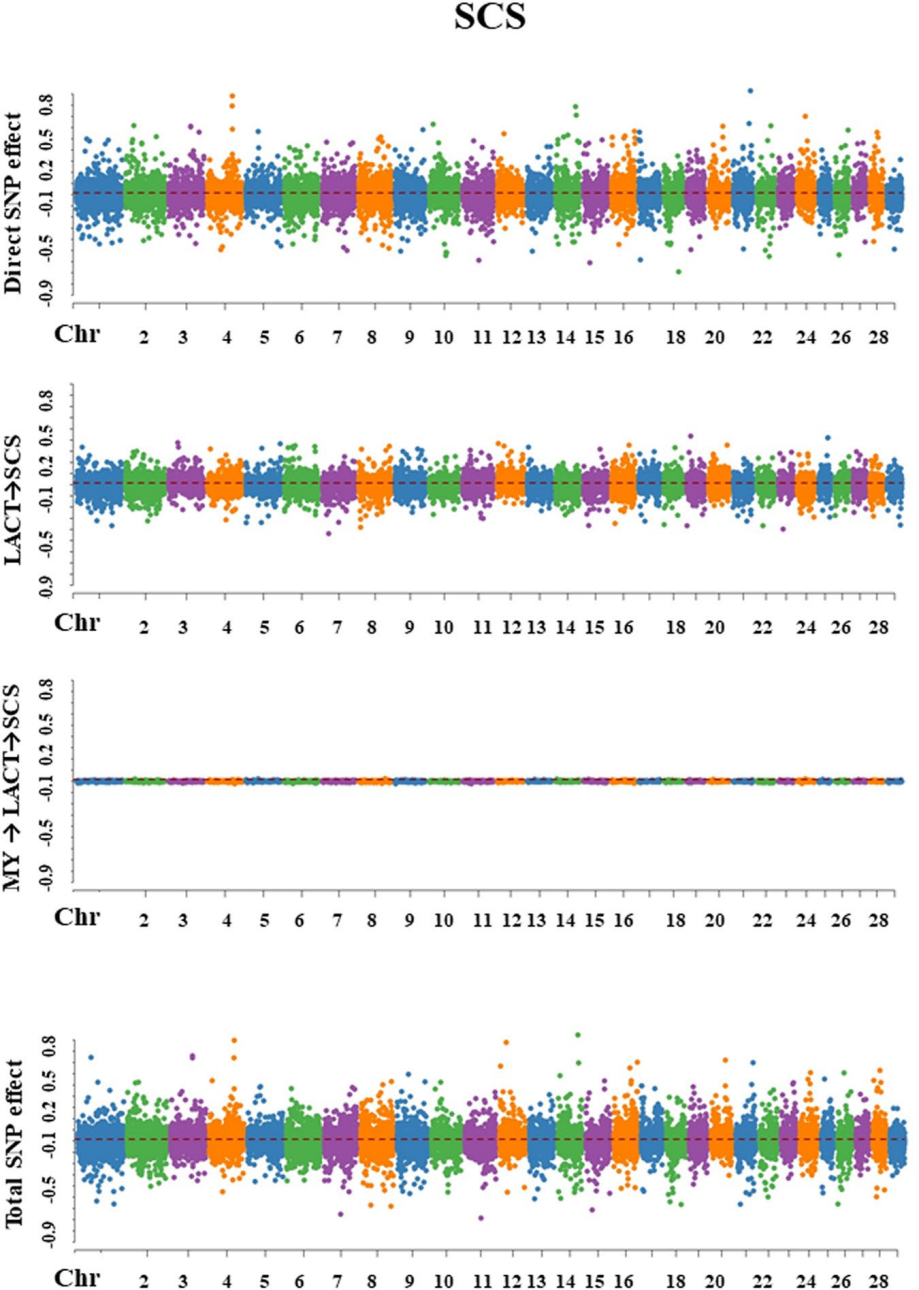
Manhattan plots for SNP effects on somatic cell score obtained using SEM-GWAS based on the network structure learned by Hill-Climbing algorithm. MY: milk yield; LACT: lactose; SCS: somatic cell score.

**Figure 6:**
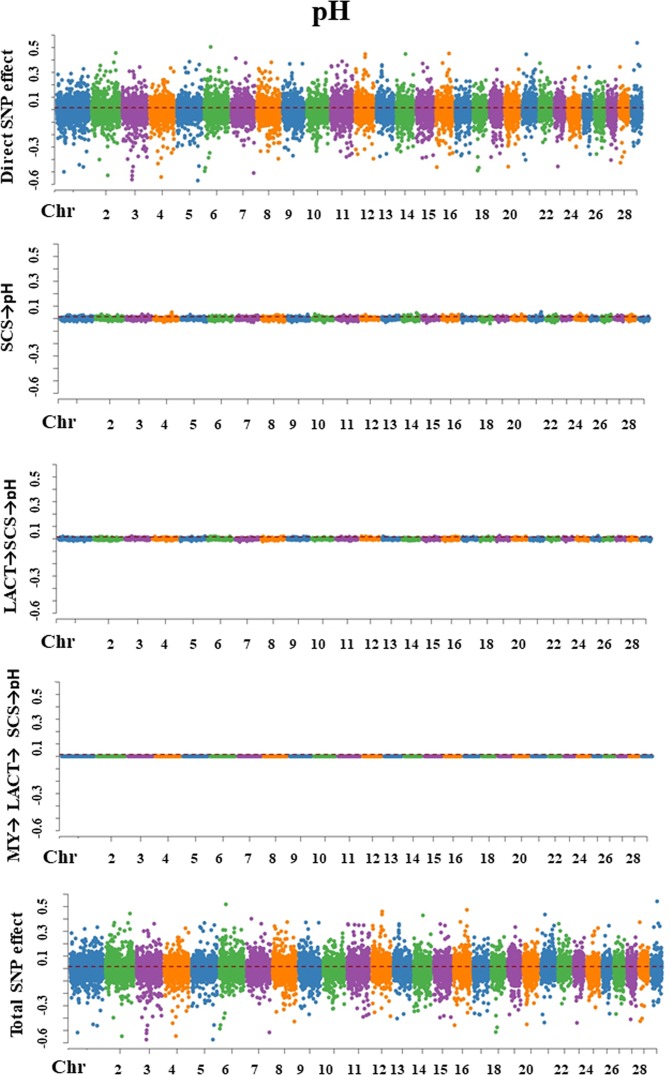
Manhattan plots for SNP effects on milk pH obtained using SEM-GWAS based on the network structure learned by Hill-Climbing algorithm. MY: milk yield; LACT: lactose; SCS: somatic cell score.

**Figure 7:**
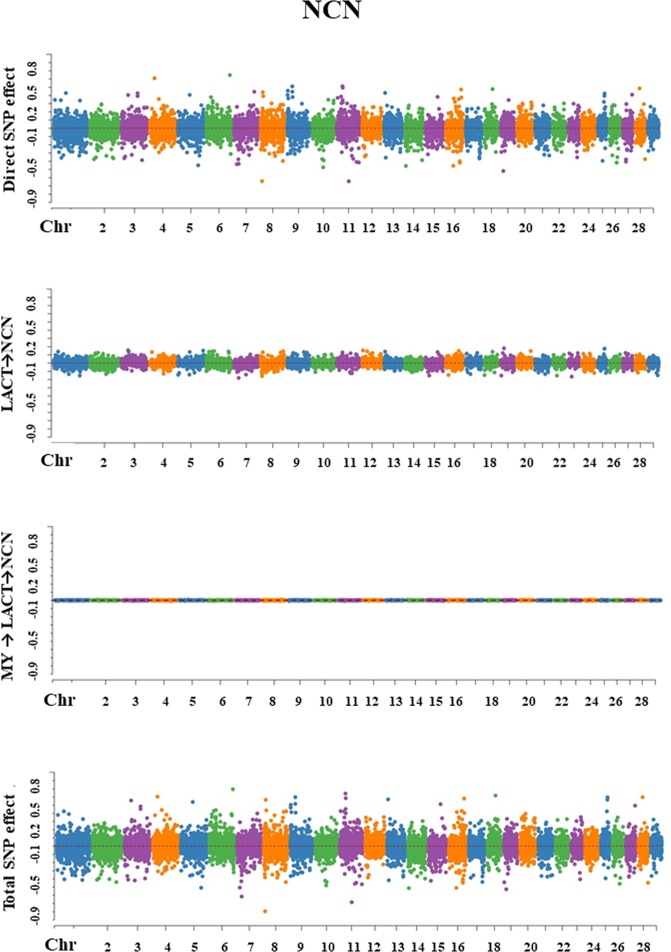
Manhattan plots for SNP effects on non-casein N obtained using SEM-GWAS based on the network structure learned by Hill-Climbing algorithm. MY: milk yield; LACT: lactose; NCN: non- casein N (expressed as % of total milk N).

For each trait, SEM-GWAS allowed us to partition SNP effects into direct and one or more indirect effects. The Manhattan plots for SNP effect decomposition are displayed in Figs. 3–7.

#### Milk yield

In the case of MY, the Bayesian network algorithm did not identify any up-stream mediator trait (Fig. 1). Therefore, the genomic architecture of MY was seemingly controlled only by direct SNP effects, i.e., the total effect of the jth SNP on MY corresponds to its own direct effect (Fig. 3).

$${{\rm{Direct}}}_{{{\rm{s}}}_{{\rm{j}}}\to {\rm{y}}{1}_{{\rm{MY}}}}={{\rm{s}}}_{{\rm{j}}({\rm{y}}{1}_{{\rm{MY}}})}$$

$${{\rm{Total}}}_{{{\rm{s}}}_{{\rm{j}}}\to {\rm{y}}{1}_{{\rm{MY}}}}={{\rm{Direct}}}_{{{\rm{s}}}_{{\rm{j}}}\to {\rm{y}}{1}_{{\rm{MY}}}}={{\rm{s}}}_{{\rm{j}}({\rm{y}}{1}_{{\rm{MY}}})}$$

#### Lactose

The overall SNP effect on LACT was decomposed into one direct effect and one indirect effect mediated by MY (MY→LACT) with a structural coefficient λ_21_(−0.065). The magnitude of this coefficient was relatively small, and so the contribution to SNP effects on LACT mediated by MY was trivial (Fig. 4).

$${{\rm{Direct}}}_{{{\rm{s}}}_{{\rm{j}}}\to {\rm{y}}{2}_{{\rm{LACT}}}}={{\rm{s}}}_{{\rm{j}}({\rm{y}}{2}_{{\rm{LACT}}})}$$

$${\rm{Indirect}}{(1)}_{{{\rm{s}}}_{{\rm{j}}}\to {\rm{y}}{2}_{{\rm{LACT}}}}={{\rm{\lambda }}}_{21}{{\rm{s}}}_{{\rm{j}}({\rm{y}}{1}_{{\rm{MY}}})}$$

$${{\rm{Total}}}_{{{\rm{s}}}_{{\rm{j}}}\to {\rm{y}}{2}_{{\rm{LACT}}}}={{\rm{Direct}}}_{{{\rm{s}}}_{{\rm{j}}}\to {\rm{y}}{2}_{{\rm{LACT}}}}+{\rm{Indirect}}{(1)}_{{{\rm{s}}}_{{\rm{j}}}\to {\rm{y}}{2}_{{\rm{LACT}}}}={{\rm{s}}}_{{\rm{j}}({\rm{y}}{2}_{{\rm{LACT}}})}+{{\rm{\lambda }}}_{21}{{\rm{s}}}_{{\rm{j}}({\rm{y}}{1}_{{\rm{MY}}})}$$

#### Somatic cell score

Overall SNP effects for SCS could be partitioned into one direct effect and two indirect effects: (1) LACT → SCS and (2) MY → LACT → SCS. LACT influenced SCS via an indirect path with structural coefficient λ_32_ (−0.632). The magnitude of this coefficient was moderate, which suggested that allelic substitutions in a QTL for LACT might affect SCS. The indirect path mediated by MY and represented by the product of the coefficients λ_21_ × λ_32_ (−0.065 × −0.632 = 0.041) gave a relatively small contribution to total SNP effects (Fig. 5).

$${{\rm{Direct}}}_{{{\rm{s}}}_{{\rm{j}}}\to {\rm{y}}{3}_{{\rm{SCS}}}}={{\rm{s}}}_{{\rm{j}}({\rm{y}}{3}_{{\rm{SCS}}})}$$

$${\rm{Indirect}}{(1)}_{{{\rm{s}}}_{{\rm{j}}}\to {\rm{y}}{3}_{{\rm{SCS}}}}={{\rm{\lambda }}}_{32}{s}_{{\rm{j}}({\rm{y}}{2}_{{\rm{LACT}}})}$$

$${\rm{Indirect}}{(2)}_{{s}_{{\rm{j}}}\to {\rm{y}}{3}_{{\rm{SCS}}}}={{\rm{\lambda }}}_{32}{{\rm{\lambda }}}_{21}{{\rm{s}}}_{{\rm{j}}({\rm{y}}{1}_{{\rm{MY}}})}$$

$$\begin{array}{ccc}{{\rm{Total}}}_{{\mathop{{\rm{s}}}\limits^{\frown {}}}_{{\rm{j}}}\to {\rm{y}}{3}_{{\rm{SCS}}}} & = & {{\rm{Direct}}}_{{{\rm{s}}}_{{\rm{j}}}\to {\rm{y}}{3}_{{\rm{SCS}}}}+{\rm{Indirect}}{(1)}_{{{\rm{s}}}_{{\rm{j}}}\to {\rm{y}}{3}_{{\rm{SCS}}}}+{\rm{Indirect}}{(2)}_{{{\rm{s}}}_{{\rm{j}}}\to {\rm{y}}{3}_{{\rm{SCS}}}}\\  & = & {{\rm{s}}}_{{\rm{j}}({\rm{y}}{3}_{{\rm{SCS}}})}+{{\rm{\lambda }}}_{32}{{\rm{s}}}_{{\rm{j}}({\rm{y}}{2}_{LACT})}+{{\rm{\lambda }}}_{32}{{\rm{\lambda }}}_{21}{{\rm{s}}}_{{\rm{j}}({\rm{y}}{1}_{{\rm{MY}}})}\end{array}$$

#### pH

For milk pH, one direct and three indirect effects (1) SCS → pH, (2) LACT → SCS → pH, and (3) MY → LACT→SCS → pH. SCS influenced milk pH via an indirect path depending on the structural coefficient λ_43_(0.057). LACT influence on pH was proportional to the product between the two coefficients λ_32_×λ_43_ (−0.632 × 0.057= −0.036). The third indirect path corresponded to the MY influence on pH which depended on the product between the three coefficients λ_21_×λ_32_×λ_43_ (−0.065 × −0.632 × 0.057=0.002). These relatively small values suggested that the contribution of SNP indirect effects to total SNP effects on milk pH was small (Fig. 6).

$${{\rm{Direct}}}_{{{\rm{s}}}_{{\rm{j}}}\to {\rm{y}}{4}_{{\rm{pH}}}}={{\rm{s}}}_{{\rm{j}}({\rm{y}}{4}_{{\rm{pH}}})}$$

$${\rm{Indirect}}{(1)}_{{{\rm{s}}}_{{\rm{j}}}\to {\rm{y}}{4}_{{\rm{pH}}}}={{\rm{\lambda }}}_{43}{{\rm{s}}}_{{\rm{j}}({\rm{y}}{3}_{{\rm{SCS}}})}$$

$${\rm{Indirect}}{(2)}_{{{\rm{s}}}_{{\rm{j}}}\to {\rm{y}}{4}_{{\rm{pH}}}}={{\rm{\lambda }}}_{43}{{\rm{\lambda }}}_{32}{{\rm{s}}}_{{\rm{j}}({\rm{y}}{2}_{{\rm{LACT}}})}$$

$${\rm{Indirect}}{(3)}_{{{\rm{s}}}_{{\rm{j}}}\to {\rm{y}}{4}_{{\rm{pH}}}}={{\rm{\lambda }}}_{43}{{\rm{\lambda }}}_{32}{{\rm{\lambda }}}_{21}{{\rm{s}}}_{{\rm{j}}({\rm{y}}{1}_{{\rm{MY}}})}$$

$$\begin{array}{ccc}{{\rm{Total}}}_{{{\rm{s}}}_{{\rm{j}}}\to {\rm{y}}{4}_{{\rm{pH}}}} & = & {{\rm{Direct}}}_{{{\rm{s}}}_{{\rm{j}}}\to {\rm{y}}{4}_{{\rm{pH}}}}+{\rm{Indirect}}{(1)}_{{{\rm{s}}}_{{\rm{j}}}\to {\rm{y}}{4}_{{\rm{pH}}}}+{\rm{Indirect}}{(2)}_{{{\rm{s}}}_{{\rm{j}}}\to {\rm{y}}{4}_{{\rm{pH}}}}+{\rm{Indirect}}{(3)}_{{{\rm{s}}}_{{\rm{j}}}\to {\rm{y}}{4}_{{\rm{pH}}}}\\  & = & {{\rm{s}}}_{{\rm{j}}({{\rm{y}}}_{4{\rm{pH}}})}+{{\rm{\lambda }}}_{43}{{\rm{s}}}_{{\rm{j}}({\rm{y}}{3}_{{\rm{SCS}}})}+{{\rm{\lambda }}}_{43}{{\rm{\lambda }}}_{32}{{\rm{s}}}_{{\rm{j}}({\rm{y}}{2}_{{\rm{LACT}}})}+{{\rm{\lambda }}}_{43}{{\rm{\lambda }}}_{32}{{\rm{\lambda }}}_{21}{{\rm{s}}}_{{\rm{j}}({\rm{y}}{1}_{{\rm{MY}}})}\end{array}$$

#### Non-casein N

Overall SNP effects on NCN were represented by one direct SNP effect and two indirect SNP effects: (1) LACT → NCN and (2) MY → LACT → NCN. LACT had an influence on NCN via an indirect with path coefficient λ_52_ (−0.262), suggesting that allelic substitutions in QTL for LACT have a moderate influence on NCN. MY contribution on the NCN total SNP effect was represented by the product of the coefficients λ_21_ × λ_52_ (−0.065 × −0.262= 0.017), and it was relatively small (Fig. 7).

$${{\rm{Direct}}}_{{{\rm{s}}}_{{\rm{j}}}\to {\rm{y}}{5}_{{\rm{NCN}}}}={{\rm{s}}}_{j({\rm{y}}{5}_{{\rm{NCN}}})}$$

$${\rm{Indirect}}{(1)}_{{{\rm{s}}}_{{\rm{j}}}\to {\rm{y}}{5}_{{\rm{NCN}}}}={{\rm{\lambda }}}_{52}{{\rm{s}}}_{{\rm{j}}({\rm{y}}{2}_{{\rm{LACT}}})}$$

$${\rm{Indirect}}{(2)}_{{{\rm{s}}}_{{\rm{j}}}\to {\rm{y}}{5}_{{\rm{NCN}}}}={{\rm{\lambda }}}_{52}{{\rm{\lambda }}}_{21}{{\rm{s}}}_{{\rm{j}}({\rm{y}}{1}_{{\rm{MY}}})}$$

$$\begin{array}{ccc}{{\rm{Total}}}_{{s}_{{\rm{j}}}\to {\rm{y}}{5}_{{\rm{NCN}}}} & = & {{\rm{Direct}}}_{{{\rm{s}}}_{{\rm{j}}}\to {\rm{y}}{5}_{{\rm{NCN}}}}+\,{\rm{Indirect}}{(1)}_{{{\rm{s}}}_{{\rm{j}}}\to {\rm{y}}{5}_{{\rm{NCN}}}}+{\rm{Indirect}}{(2)}_{{{\rm{s}}}_{{\rm{j}}}\to {\rm{y}}{5}_{{\rm{NCN}}}}\\  & = & {{\rm{s}}}_{{\rm{j}}({\rm{y}}{5}_{{\rm{NCN}}})}+{{\rm{\lambda }}}_{52}{{\rm{s}}}_{{\rm{j}}({\rm{y}}{2}_{{\rm{LACT}}})}+{{\rm{\lambda }}}_{52}{{\rm{\lambda }}}_{21}{{\rm{s}}}_{{\rm{j}}({\rm{y}}{1}_{{\rm{MY}}})}\end{array}$$

We compared the direct and indirect SNP effects with the total effects for pH, LACT, SCS, and NCN. Direct SNP effects were positively and highly correlated (R^2^ > 0.90) with total SNP effects for all traits except for SCS (R^2^ = 0.64). The correlation between all indirect SNP effects on pH and LACT with total SNP effects was close to 0 (Supplementary Figures S1–S2) as well as between MY → LACT → SCS and MY → LACT → NCN effects and total SNP effects (Supplementary Figures S3–S4). Weak positive correlations with total SNP effects were found between LACT → SCS and LACT → NCN with total SNP effects (Supplementary Figures S3–S4).

### Joint use of MTM-GWAS and SEM-GWAS

Since MTM-GWAS is a well-recognized approach when dealing with multiple correlated phenotypes, we assessed the agreement between SNP effects derived from MTM-GWAS and overall SNP effects from SEM-GWAS. A high agreement (R^2^ > 0.85) was found for all the traits investigated between SNP effects detected with the two models which are based on the same multivariate approach and same covariances matrices (Supplementary Figure S5).

The power of SEM-GWAS is the potential to partition the source of SNP effects, rather than partitioning total standard errors or *P*-values. Therefore, we used MTM-GWAS solutions to declare significant associations. No significant SNP was found for the five traits at genome-wide significance log10(*P*) threshold (5.355). Six significant SNP were identified for SCS at suggestive significance log10(*P*) threshold (4.054) (Table 3, Supplementary Figure S1). Five SNP were detected on BTA4 at ~ 72.99 and at ~76.31–79.92 Mb and one on BTA13 at ~53.80 Mb. The effects decomposition for these SNP is provided in Table 3. Results showed that the path LACT → SCS provided a relevant positive contribution to total SNP effects by increasing the direct SNP effect from 0.020 (rs110854438 and rs110811284) to 0.062 (rs41569794). On the other hand, the effect size of the path MY → LACT → SCS was very small (0.001–0.003 standard deviations) with negative values for rs41569794, rs110736919, rs41615292, rs110854438 and rs110811284, and positive for rs110490432.

**Table 3:** Effect decomposition provided by SEM-GWAS for the significant SNP identified by MTM-GWAS.

SNP	CHR	BP	Direct effect^a^	LACT → SCS^b^	MY → LACT → SCS^c^	Total effect^d^	−log10Pvalue
rs41569794	4	72532921	0.135	0.062	−0.003	0.194	4.656
rs110736919	4	76247713	0.171	0.028	−0.002	0.197	4.494
rs41615292	4	79930421	0.157	0.026	−0.001	0.182	4.461
rs110854438	4	76312755	0.173	0.020	−0.002	0.191	4.377
rs110811284	4	76377517	0.173	0.020	−0.002	0.191	4.374
rs110490432	13	5380158	0.158	0.040	<0.001	0.198	4.054

CHR: chromosome; BP: SNP location in bp; LACT: lactose; SCS: somatic cell score; MY: milk yield.

^a^Direct effect of SNP on SCS.

^b^Indirect effect on SCS mediated by LACT.

^c^Indirect effect on SCS mediated by MY and LACT.

^d^Sum of direct and indirect SNP effect.

### Pathway enrichment analyses

**Figure 8:**
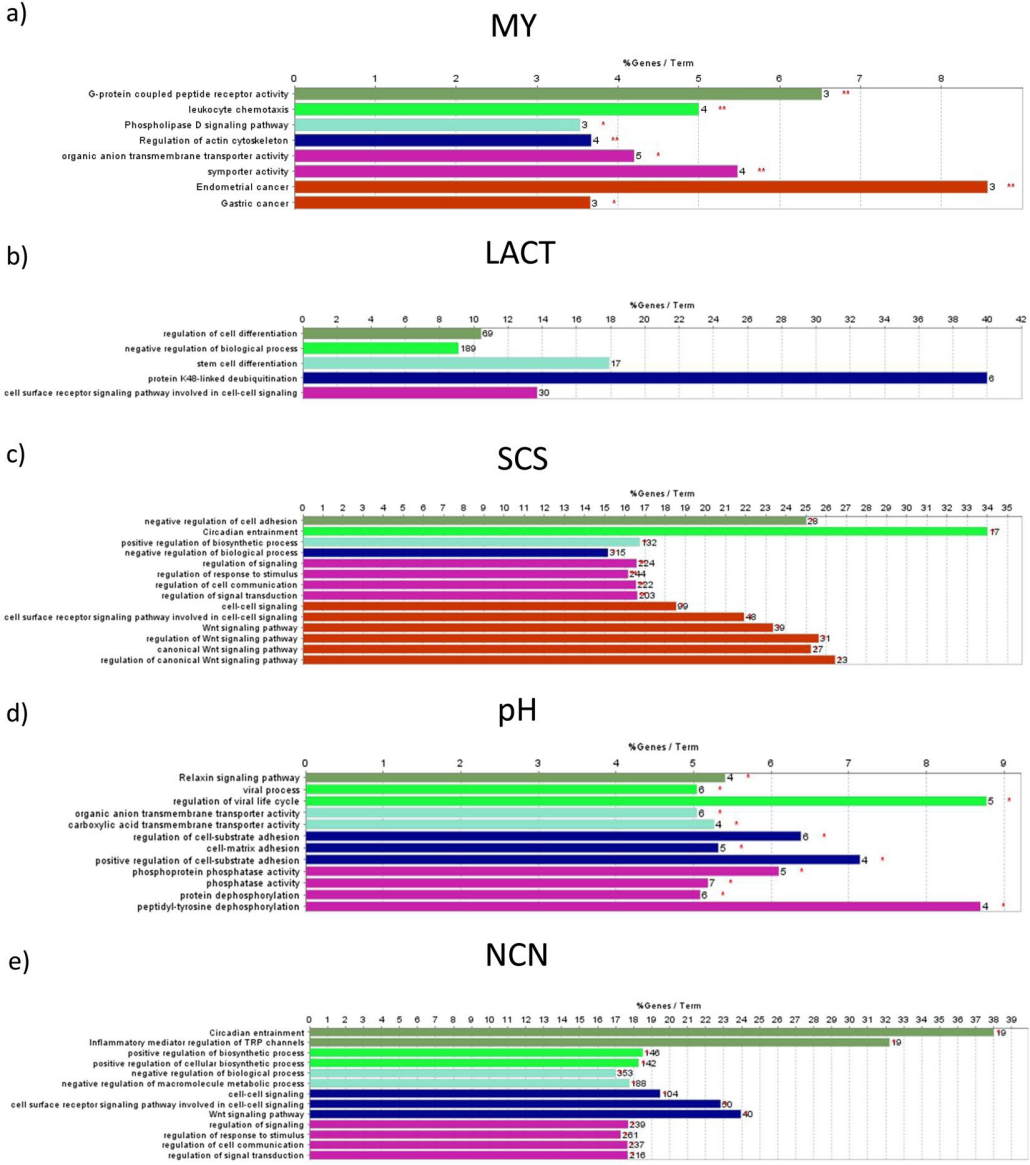
Significantly enriched GO terms and KEGG pathways for the investigated traits. (**a**) Milk yield (MY); (**b**) Lactose (LACT); (**c**) Somatic cell score (SCS); (**d**) Milk pH (pH); (**e**) Non-casein N (NCN, expressed as % of total milk N). SNPs obtained from MTM-GWAS (*P* < 0.05) were mapped to genes based on 15 kb distance from the coding region using the biomaRt R package^40,41^. The Cytoscape plugin Cluego^43^ was used to identify overrepresented pathways and GO terms based on a right-sided hypergeometric test with false discovery rate set at 0.05.

Several ontologies and pathways were enriched (FDR < 0.05) for the traits investigated (Fig. 8). For instance, pathways connected with membrane transport activity were identified for MY and pH, such as organic anion transmembrane transporter activity (both MY and pH; FDR = 0.0118 and 0.0144, respectively), symporter activity (MY, FDR = 0.0068) and carboxylic acid transmembrane transporter activity (pH, FDR = 0.0277). The associated genes included *SLC13A5, SLC4A4, SLC5A10*, and *SLC16A11*, which are involved in the transport of citrate, sodium bicarbonate and sodium/glucose and monocarboxylic acid. We also found a functional link between MY and phospholipase D signaling pathway (3 genes, including *DGKE*; FDR = 0.0220), G-protein coupled peptide receptor activity (3 genes; FDR = 0.0065) and leukocyte chemotaxis (4 genes; FDR = 0.0048). A functional group including protein dephosphorylation (6 genes; FDR = 0.0161), peptidyl-tyrosine dephosphorylation (4 genes; FDR = 0.0136) and phosphatase activity (7 genes; FDR = 0.0156) was associated to milk pH. Several pathways and ontologies functionally connected with signal transduction and cell-cell signaling were enriched for milk components. In particular, the cell surface receptor signaling pathway was overrepresented for LACT (30 genes; FDR = 0.0327), SCS (48 genes; FDR = 0.0214), and NCN (50 genes; FDR = 0.0382). Moreover, the Wnt signaling was enriched for SCS (39 genes; FDR = 0.0195) and NCN (40 genes; FDR = 0.0419). Finally, 188 genes (including *CSN1S1, ACACB, PLCB1, PRKCG, PPARG*, and *TSC1*) belonging to negative regulation of macromolecule metabolic process (FDR = 0.0363) were associated to NCN.

## Discussion

High MY is known to increase risk of clinical mastitis, and consequently increase SCS, which is an indicator of mastitis. Mastitis, in turn, will reduce MY in the remaining portion of lactation. Such relationships have been described previously by a model with a recursive effect from SCS to MY providing evidence for the possible existence of unfavorable effects between MY and SCS^26^. In this study, we explored the existence of putative dependencies among a set of five phenotypes related to udder health, including not only MY and SCS but also milk pH, LACT, and NCN, which are related to udder health status. The network structure identified by the HC Bayesian network algorithm was incorporated into a SEM-based GWAS model to decompose SNP effects into direct effects on the trait and effects mediated by up-stream traits in the phenotypic network.

Average phenotypic values obtained for MY, LACT, pH and SCS were in line with previous results reported for Brown Swiss cattle breed^27,28^. Regarding NCN, this trait corresponds to the sum of whey proteins and non-protein N percentages (i.e., complement of casein index to 100%). Average values obtained in this study for NCN (21.96 ± 1.23%) are therefore coherent with values reported in the literature for casein index in Brown Swiss (76.76 ± 2.58%)^29^.

Genomic heritabilities estimated by the multi-trait Bayesian GBLUP for the investigated traits were in the range of genetic values obtained with univariate models^27,30–32^, except for milk pH, which showed higher values. The only statistically relevant genomic correlation was found between LACT (an udder health indicator trait) and NCN (−0.280), which might support the hypothesis that non-protein N in milk might be considered as an indicator of mammary gland inflammation. Additionally, we previously found negative genetic correlations between serum proteins (albumins and globulins) and lactose percentage^33^.

The application of the HC algorithm allowed us to infer the network structure from a residual covariance matrix, after accounting for polygenic additive effects. New insights were provided on the relationships among traits related to udder health in dairy cattle. In particular, we found a putative mediation of LACT on SCS and NCN in our phenotypic network, which is supported by the magnitude of the path coefficients reflecting the strength of the dependency relationship. The relationships between lactose in milk and udder health or SCC have been widely investigated, and lactose percentage in the milk of mastitic animals was significantly below the average values of the healthy animals^34^. Lactose is a β-galactoside consisting of galactose and glucose residues, and it is the main carbohydrate in mammalian breast milk. Galectins are a family of proteins that bind specifically to β-galactosides such as lactose and have regulatory functions in the immune system. The interaction of lactose with particular galectin members seemed to largely determine its anti- or pro- inflammatory effects^35^. It is possible that the leakage of lactose from milk to blood might have a chemotactic effect attracting macrophages and leukocytes from the blood to the mammary gland. This might also explain the negative sign of the path coefficient (−0.632). Monocyte and macrophages migrating to the inflammation site secrete inflammatory mediators, including proteinases such as plasminogen activators which can increase the level of plasmin activity in mastitic milk^36^. This might also support the role of LACT as an up-stream trait to NCN and the negative sign of the path coefficient (−0.262). On the other hand, the strength of edges between milk pH and the other traits in the network suggested this trait had a weak connectivity with MY, LACT and NCN. The only exception was the path between SCS and pH, which had strength values > 50%. Therefore, we used prior biological information to support the existence of a relationship between these traits and infer its possible direction. It is well-recognized that milk pH increases with elevated SCC due to changes in milk ionic equilibrium which results from the mammary tissue injury^23,37^. On the other hand, a possible dependency relationship with direction pH → SCS seemed to be much more difficult to envisage. However, the coefficient for this path (λ_43_=0.057) was small, suggesting that the role of SCS in mediating SNP effects on milk pH is marginal.

By jointly applying MTM-GWAS and SEM-GWAS, we identified 6 suggestive significant SNP for SCS and we quantified the contribution of MY and LACT acting as mediator traits to total SNP effects. In particular, the contribution of the indirect path MY → LACT → SCS to total SNP effect on SCS was very small while LACT revealed to be a relevant mediator trait affecting total SNP effects, especially for marker rs41569794. Interestingly, marker rs41569794 mapped on BTA4 at ~0.1 Mb from *STEAP4*, which is a metalloreductase involved in the control of systemic metabolic homeostatis by integrating inflammatory and metabolic responses. This gene was among the top 10 genes with the greatest increase in expression in milk somatic cells after intra-mammary infection with *S. aureus* in goat^38^. The marker rs110736919 corresponded to an intron variant of *ADCY1*. This gene codes for a calmodulin-sensitive adenylyl cyclase. According to GO annotations, *ADCY1* seemed to have a role in several processes such as regulation of circadian rhythm, cellular response to calcium ion and neuroinflammatory response. We did not find any association between this gene and mastitis or mammary gland inflammation. However, SNP within or close to *ADCY1* have been associated to fertility traits in dairy cows^39,40^. The marker rs41615292 corresponded to an intergenic variant, which mapped close to *INHBA* (~0.6 Mb). The INHBA is a member of the TGF-β superfamily^41^, which has a role in apoptosis. The damage induced by mastitis to the mammary tissue can be induced by apoptosis or necrosis^42^. Accordingly, Fonseca *et al*.^43^ found an increase in the expression of this gene in bovine mammary gland after experimental infection with *Streptococcus agalactiae*. The markers rs110854438 and rs110811284 mapped close to *ADCY1* (~40 Kb) and at ~0.2 Mb from *IGFBP3* and *IGFBP1*. It has been shown that lactoferrin specifically binds to extracellular IGFBP3 and plays a key role in the entry of IGFBP3 into mammary cells nucleus^44^. Lactoferrin has a well-known role in the modulation of inflammatory process since it prevents the release of cytokines from monocytes and regulates the proliferation and differentiation of immune cells^45^. Therefore, it has been considered as a good indicator of mastitis in dairy cows similarly to SCC^46^. Finally, the marker rs110490432 corresponded to an intergenic variant which mapped close (at ~76 Kb) to *BTBD3*. To our knowledge, no association of this gene to immune response has been previously demonstrated in dairy cows.

Pathways enrichment analyses showed that variants associated to MY, LACT, SCS, pH, and NCN aggregated in various biological pathways, which were frequently shared among traits as further support for their intercorrelation. The secretion of milk depends on the activity of several membrane transport systems on mammary secretory cells^47^. Moreover, Na^+^/H^+^ exchange and Na^+^/HCO3^-^ cotransport is involved in the regulation of mammary cell pH^48^. This might explain the overrepresentation of pathways connected with membrane transport activity for both MY and pH. Genes involved in the Wnt signaling were associated to both SCS and NCN. Inflammatory epithelial cells were shown to induce changes in stromal fibroblast characteristics in bovine mammary gland with mastitis which are mediated by Wnt signal pathway components^49^. A clear connection between Wnt signaling and milk protein metabolism was not found. However, among the associated genes, there was *GSKIP* which is a negative regulator of GSK3β in the Wnt signaling pathway. Recently, it was reported that mTORC1 inhibition of GSK3β regulates the production of pro- and anti-inflammatory cytokines, which is in line with the involvement of Wnt sinaling in the control of inflammation. However, mTOR signaling pathway plays also an important role in milk protein synthesis^50^. Regulation of signaling pathway for LACT might further support for its putative chemotactic activity towards immune cells.

This study represents the first application of SEM-GWAS in dairy cattle, in particular, to udder health traits. We showed that SEM is a flexible approach which allows to model the relationships among SNP and phenotypes including the contribution of potential mediator traits, which might be particularly useful especially in the case of highly interconnected phenotypes contributing to a final outcome through common or different pathways^51^. SEM-GWAS might be therefore considered as an extension of MTM-GWAS which accounts for the network structure among phenotypes and is able to identify the contribution of direct and indirect effects on the total SNP effect. Caution must be used, however, when interpreting SEM as a causal model even if it is corroborated by the data, since causal pathways may be difficult to model in a multi‐trait framework in which many genes and interactions are involved^52^. Moreover, it is clear that omitting variables implicated in causal processes may distort views of the system^53^.

The identification of indirect effects can provide a better understanding of outcome mechanisms and help in designing selection strategies and management decisions aimed at improving udder health in dairy cattle. Our results need to be validated on a larger population and using a database including repeated records along the lactation, together with records of clinical mastitis cases which might improve the accuracy for the detection of putative causal paths among the traits investigated.

## Methods

### Ethics statement

This study did not require any specific ethics permit. The cows sampled belonged to commercial private herds and were not experimentally manipulated. Milk samples were collected during routine milk recording coordinated by technicians from the Breeders Association of Trento Province (Italy).

### Phenotypes and genotypes

Individual milk samples were collected from 1,264 Italian Brown Swiss cows in commercial herds located in the Alpine province of Trento (Italy). Details of the animals used in this study and the characteristics of the area are reported in Bittante *et al*.^54^. Briefly, samples were obtained from cows reared in 85 herds with parities of 1 to 5 and days in milk ranging from 5 to 449. Individual milk samples were collected once during the evening milking and immediately refrigerated at 4 °C (without any preservative). One sub-sample (50 mL; destined for milk composition analysis) was transported to the Milk Quality Laboratory of the Trento Breeders Association. The second subsample (about 2,000 mL) was transferred to the Milk Laboratory of the Department of Agronomy, Food, Natural Resources, Animals and Environment (DAFNAE) at the University of Padova (Legnaro, Padova, Italy)^55^. A detailed description of the genetic structure and connections between animals has been previously reported^56^.

The pH analysis was carried out using a Crison Basic 25 electrode (Crison, Barcelona, Spain). Somatic cell count values were determined by a Fossomatic FC counter (Foss) and SCS were obtained through logarithmic transformation [log2(SCC/100,000) + 3]^57^. An aliquot of each milk sample was analyzed for fat, protein, casein, and lactose (%) using a Milkoscan FT6000 (Foss Electric A/S, Hillerød, Denmark) at the Milk Laboratory of the Department of Agronomy, Food, Natural Resources, Animals and the Environment (DAFNAE), the University of Padua (Italy). NPN was calculated as the differences between total milk N and casein N and expressed as a percentage.

The Illumina BovineSNP50 v.2 BeadChip (Illumina Inc., San Diego, CA) was used to genotype 1,152 cows (blood samples were not available for all the phenotyped animals). Quality control excluded markers with call rates <95%, minor allele frequencies <0.01 and extreme deviation from the Hardy-Weinberg equilibrium (*P* < 0.001, Bonferroni corrected). Missing genotypes were imputed by the Beagle software Version 3.3.2^58^. After filtering, 1,011 cows and 37,519 SNPs were retained for subsequent analyses.

### Multi-trait genomic best linear unbiased prediction

A Bayesian multi-trait genomic best linear unbiased prediction (GBLUP) model was fitted to five udder health-related traits (i.e., MY, LACT, SCS, pH, and NCN) using the R package MTM (https://github.com/QuantGen/MTM) to obtain posterior means of model residuals as input for inferring putative dependencies among traits according to the model:

1 $${\bf{y}}={\bf{X}}{\bf{b}}+{\bf{Z}}{\bf{g}}+{\bf{e}},$$

where **y** is the vector of phenotypes (t = 5), **X** is the t × k incidence matrix of non-genetic effects; **b** is the k × 1 vector of the non-genetic effects including the intercept and the following: (i) days in milk of the cow (classes of 30 days each), (ii) parity of each cow (classes of 1, 2, 3, ≥ 4), and (iii) herd-date effect (85 levels); **Z** is the n × m incidence matrix relating animals with additive genomic effects; **g** is the m × 1 vector of additive genomic effects, and **e** is the t × 1 vector of residuals. The **g** and **e** vectors were assumed to follow the independent multivariate Gaussian distributions ${\bf{g}} \sim {\rm{N}}(0,\,{\varSigma }_{g}\otimes {\bf{G}})$ and ${\bf{e}} \sim {\rm{N}}(0,{\varSigma }_{{\boldsymbol{e}}}\otimes {\bf{I}})$, respectively, where **G** is the genomic relationship matrix for genetic effects, **I** is the identity matrix for residuals, **Σ**_**g**_ and **Σ**_**e**_ are the t × t variance-covariance matrices of genetic effects and residuals, respectively. Here, ⊗ indicates the Kronecker product. The **G** matrix was computed as ${\bf{W}}{\bf{W}}{\prime} /2\sum {p}_{j}(1-{p}_{j})$, where **W** is an n × m matrix of centered SNP genotypes having values of 0−2*p*_*j*_ for zero copies of the reference allele, 1−2*p*_*j*_ for one copy of the reference allele, and 2−2*p*_*j*_ for two copies of the reference allele^59^. Here, *p*_*j*_ corresponds to the allele frequency at SNP *j* =1, …, *m*. Flat priors were assigned to the intercepts and to the vector of fixed effects. Independent multivariate normal priors with null mean and inverse Wishart distributions for covariances matrices were assigned to the vectors of random additive genomic effects and residual effects.

Marginal posterior distributions were obtained using a Markov chain Monte Carlo (MCMC) approach with Gibbs sampling. We used a burn-in of 20,000 and from 150,000 MCMC samples we retained 75,000 MCMC samples (thin interval =2). Chain lengths and burn-in period were assessed from visual inspection of the trace plots. Posterior means were used as point estimates for all parameters. Lower and upper bounds of the highest 95% probability density regions (HPD 95%) were obtained from the estimated marginal densities. For the correlations, in addition to the means of each marginal posterior distribution, we also estimated the probability of each mean being greater than 0 when the mean was positive, or lower than 0 when the mean was negative (*P*). All estimates with *P* greater than 95% were considered “relevant” correlations.

### Bayesian networks

A Bayesian network is a probabilistic graphical model where nodes represent the phenotypes and edges represent probabilistic dependencies between them; the absence of an edge implies conditional independence between variables^60^. We used the score-based algorithm Hill-Climbing (HC)^61^ implemented in the R package bnlearn^62^ to infer the structure of the Bayesian residual phenotypic network for the five udder health traits. We also computed the change in Bayesian information criterion (BIC) score after each edge removal in the algorithm to infer their relative contribution to the overall BIC score of the network. The edge strength and uncertainty of direction were estimated by a bootstrapping procedure (n = 50,000 bootstrap samples) as described in Scutari and Denis^63^. An edge strength> 85% was used to select only high-confidence relationships.

### Multi-trait GWAS

MTM-GWAS analyses were conducted using the “SNP Snappy” strategy^64^ implemented in the mixed model package WOMBAT^65^, according to the following model, which did not consider the inferred network structure:

2 $${\bf{y}}={\bf{W}}{\bf{s}}+{\bf{X}}{\bf{b}}+{\bf{Z}}{\bf{g}}+{\bf{e}},\,$$

where **y** is the vector of scaled phenotypes (t = 5), **W** is the n × t by t matrix of genotype codes of SNP marker j, **s** is the t × 1 vector of additive effects for SNP marker j, and other terms were previously described. Variance-covariance structures were assumed the same as for Eq. (1). We fitted MTM-GWAS for each SNP individually to obtain the following vector of marker estimates for each trait: ${\bf{s}}=[{{\rm{s}}}_{{\rm{MY}}},{{\rm{s}}}_{{\rm{LACT}}},{{\rm{s}}}_{{\rm{SCS}}},{{\rm{s}}}_{{\rm{pH}}},{{\rm{s}}}_{{\rm{NCN}}}]$. A t statistic was used to obtain *P*-values: ${{\rm{T}}}_{{\rm{ij}}}={{\rm{s}}}_{{\rm{j}}}/{\rm{se}}({{\rm{s}}}_{{\rm{j}}})$, where s is the point estimate of the jth SNP effect and se(s_j_) is its standard error. The *P* value threshold for declaring significant associations was determined calculating the effective number of independent tests according to Li and Ji^66^ using the R package poolR. A genome-wide significant threshold of log10(*P*)= 5.355 (0.05/11315) and a suggestive significant threshold of log10(*P*)=4.054 (1/11315) were adopted.

### Structural equation model for GWAS

SEM –GWAS analyses were conducted using the SNP Snappy strategy^64^ implemented in the mixed model package WOMBAT^65^. The SEM model described in Gianola and Sorensen^3^ was extended for GWAS according to Momen *et al*.^11,67^:

3 $${\bf{y}}={\boldsymbol{\Lambda }}{\bf{y}}+{\bf{W}}{\bf{s}}+{\bf{X}}{\bf{b}}+{\bf{Z}}{\bf{g}}+{\bf{e}},$$

where **y** is the vector of scaled phenotypes (t = 5) and **Λ** is a t × t matrix of regression coefficients (structural coefficients) based on the learned structure from the Bayesian network using the residuals:

$${\boldsymbol{\Lambda }}=[\begin{array}{ccccc}0 & 0 & 0 & 0 & 0\\ {{\rm{\lambda }}}_{{\rm{MY}}\to {\rm{LACT}}} & 0 & 0 & 0 & 0\\ 0 & {{\rm{\lambda }}}_{{\rm{LACT}}\to {\rm{SCS}}} & 0 & 0 & 0\\ 0 & 0 & {{\rm{\lambda }}}_{{\rm{SCS}}\to {\rm{pH}}} & 0 & 0\\ 0 & {{\rm{\lambda }}}_{{\rm{LACT}}\to {\rm{NCN}}} & 0 & 0 & 0\end{array}]$$

The vectors **g** and **e** were assumed to have a joint distribution $$[\begin{array}{c}{\boldsymbol{g}}\\ {\boldsymbol{e}}\end{array}]={\boldsymbol{N}}\{[\begin{array}{c}0\\ 0\end{array}],[\begin{array}{cc}{\varSigma }_{{\boldsymbol{g}}}\otimes \,{\bf{G}} & 0\\ 0 & {\boldsymbol{\Psi }}\end{array}]\}$$, and the residual covariance matrix was diagonal, with $$\varPsi =[\begin{array}{ccccc}{\sigma }_{e(MY)}^{2} & 0 & 0 & 0 & 0\\ 0 & {\sigma }_{e(LACT)}^{2} & 0 & 0 & 0\\ 0 & 0 & {\sigma }_{e(SCS)}^{2} & 0 & 0\\ 0 & 0 & 0 & {\sigma }_{e(pH)}^{2} & 0\\ 0 & 0 & 0 & 0 & {\sigma }_{e(NCN)}^{2}\end{array}]$$.

The other terms were defined previously.

We considered SEM residuals to be independent within individual, which is required to make the structural coefficients likelihood-identifiable. The structural coefficients represented the effect size of edges between phenotypes in the Bayesian network and they were used to develop a set of structural equations to factorize total SNP effects into direct and indirect components.

The important difference with respect to MTM in Eq. (2) is that in SEM, the effect of a SNP on phenotype is not considered as the overall effect, but only as a direct effect. Indirect effects for the same SNP are those mediated by up-stream traits in the phenotypic network. Indirect effects are computed by multiplying path coefficients for each path linking the SNP to an associated variable, and then summing over all such paths^68,69^. The overall effect of a SNP on a specific trait is then the sum of all direct and indirect effects contributing to that trait.

### Pathway enrichment analyses

Pathway enrichment analyses were carried out to identify weaker but related signals which were missed by GWAS analysis due to the stringency in *P*-value thresholds. The assumption is that these signals might be associated with genes participating in organized pathways or biological functions. We first selected “relevant” SNP from the MTM-GWAS results based on a nominal *P*-value (<0.05). Then, we used the R package biomaRt^70,71^ for mapping SNP to genes based on a distance of 15 kb according to the Ensembl *Bos taurus* UMD3.1 assembly^72^. For each trait, we carried out functional enrichment analyses on the list of significant genes using the Cytoscape plugin ClueGo^73^ to identify significantly overrepresented pathways and ontologies (right-sided hypergeometric enrichment test, false discovery rate <0.05). We used all the SNP/genes in the chip as a background.

## Supplementary information

Supplementary information.

## Data Availability

Data is available by contacting the corresponding author.
