# Supplementary material for: Structural equation modeling for investigating multi-trait genetic architecture of udder health in dairy cattle
Source: Sci Rep. 2020 May 8;10:7751. doi: 10.1038/s41598-020-64575-3 (PMC7210309; doi:10.1038/s41598-020-64575-3)
Supplement: Supplementary file 1 — Supplementary information. [file 41598_2020_64575_MOESM1_ESM.docx]

**Structural equation modeling for investigating multi-trait genetic architecture of udder health in dairy cattle**

**Sara Pegolo^1*^, Mehdi Momen^2^, Gota Morota^2^, Guilherme J. M. Rosa^3,4^, Daniel Gianola^3,5^, Giovanni Bittante^1^ & Alessio Cecchinato^1^**

^1^Department of Agronomy, Food Natural resources, Animals and Environment, University of Padua, Legnaro (PD), Italy

^2^Department of Animal and Poultry Sciences, Virginia Polytechnic Institute and State University, Blacksburg, VA, USA

^3^Department of Animal Sciences, University of Wisconsin, Madison, WI, USA

^4^Department of Biostatistics and Medical Informatics, University of Wisconsin, Madison, WI, USA

^5^Department of Dairy Science, University of Wisconsin, Madison, WI, USA

*Corresponding author: [sara.pegolo@unipd.it](mailto:sara.pegolo@unipd.it)

**Supplementary material S1.** Set of structural equations used to estimate SEM parameters and SNP effects.

$${y_{1}}_{\mathrm{MY}} ={\mu+W}_{\mathrm{ij}}s_{j{(y1}_{\mathrm{MY}})}+X_{1}b_{1}+ Z_{1}g_{1}+e_{1}$$

$${y_{2}}_{\mathrm{LACT}}={\mu+\lambda_{21}y_{1_{MY}}+ W}_{\mathrm{ij}}s_{j{(y2}_{\mathrm{LACT}})}+X_{2}b_{2}+Z_{2}g_{2}+e_{2}$$

$$=\mu+\lambda_{21}\left[ W_{\mathrm{ij}}s_{j{(y1}_{\mathrm{MY}})} +X_{1}b_{1}+Z_{1}g_{1}+e_{1} \right]+W_{\mathrm{ij}}s_{j{(y2}_{\mathrm{LACT}})}+{X_{2}b_{2}+Z}_{2}g_{2}+e_{2}$$

$${y_{3}}_{\mathrm{SCS}}={\mu+\lambda_{32}y_{2_{\mathrm{LACT}}}+ W}_{\mathrm{ij}}s_{j{(y3}_{\mathrm{SCS}})}+{X_{3}b_{3}+Z}_{3}g_{3}+e_{3}$$

$$={\mu+\lambda}_{32}\left[ \lambda_{21}\left[ W_{\mathrm{ij}}s_{j{(y1}_{\mathrm{MY}})} +X_{1}b_{1}+Z_{1}g_{1}+e_{1} \right]+W_{\mathrm{ij}}s_{j{(y2}_{\mathrm{LACT}})}+{X_{2}b_{2}+Z}_{2}g_{2}+e_{2} \right]+W_{\mathrm{ij}}s_{j{(y3}_{\mathrm{SCS}})}+X_{3}b_{3}+Z_{3}g_{3}+e_{3}$$

$${y_{4}}_{\mathrm{pH}} =\mu+\lambda_{43}{y_{3}}_{\mathrm{SCS}}{+ W}_{\mathrm{ij}}s_{j{(y4}_{\mathrm{pH}})}+X_{4}b_{4}+Z_{4}g_{4}+e_{4}$$

$$=\mu+\lambda_{43}\left[ \lambda_{32}\left[ \lambda_{21}\left[ W_{\mathrm{ij}}s_{j{(y1}_{\mathrm{MY}})} +X_{1}b_{1}+Z_{1}g_{1}+e_{1} \right]+W_{\mathrm{ij}}s_{j{(y2}_{\mathrm{LACT}})}+{X_{2}b_{2}+Z}_{2}g_{2}+e_{2} \right]+W_{\mathrm{ij}}s_{j{(y3}_{\mathrm{SCS}})}+X_{3}b_{3}+Z_{3}g_{3}+e_{3} \right]+W_{\mathrm{ij}}s_{j{(y4}_{\mathrm{pH}})}+{X_{4}b_{4}+Z}_{4}g_{4}+e_{4}$$

$${y_{5}}_{\mathrm{NCN}}=\mu+\lambda_{52}y_{2_{\mathrm{LACT}}}{+ W}_{\mathrm{ij}}s_{j{(y5}_{\mathrm{NCN}})}+{X_{5}b_{5}+Z}_{5}g_{5}+e_{5}$$

$$=\lambda_{52}\left[ \lambda_{21}\left[ W_{\mathrm{ij}}s_{j{(y1}_{\mathrm{MY}})} +X_{1}b_{1}+Z_{1}g_{1}+e_{1} \right]+W_{\mathrm{ij}}s_{j{(y2}_{\mathrm{LACT}})}+{X_{2}b_{2}+Z}_{2}g_{2}+e_{2} \right]+W_{\mathrm{ij}}s_{j{(y5}_{\mathrm{NCN}})}+{X_{5}b_{5}+Z}_{5}g_{5}+e_{5}$$

where **y** the vector of scaled phenotype, **W** is the n ×m matrix of genotype codes, **s**_j_ is the vector of the additive effect of jth SNP, **µ** is the intercept; **X** is the incidence matrix of non-genetic effects; **b** is the vector of the non-genetic effects of i) days in milk of the cow (classes of 30 days each), ii) parity of each cow (classes of 1, 2, 3, ≥ 4), and iii) herd-date effect (85 levels); **Z** is the incidence matrix relating animals with additive genetic effects; **g** is the vector of additive genetic effects, and **e** is the vector of residuals; $\lambda_{21}$ is the structural coefficient for the path MY→LACT; $\lambda_{32}$ is the structural coefficient for the path LACT→SCS; $\lambda_{43}$ is the structural coefficient for the path SCS→pH; $\lambda_{52}$ is the structural coefficient for the path LACT→NCN.


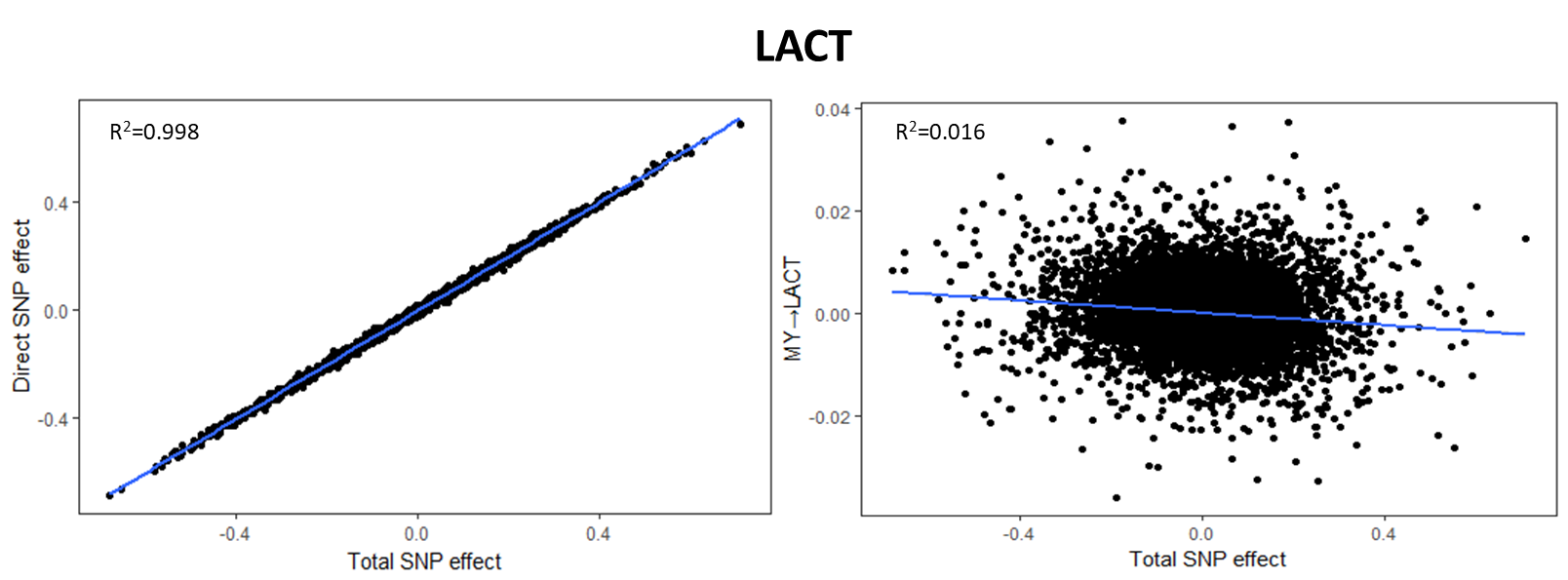


**Supplementary Figure S1.** **Scatter plots of decomposed SNP effects for milk lactose percentage.** Each point corresponds to the estimated effect of a SNP which affects lactose (LACT) directly or indirectly; MY: milk yield.


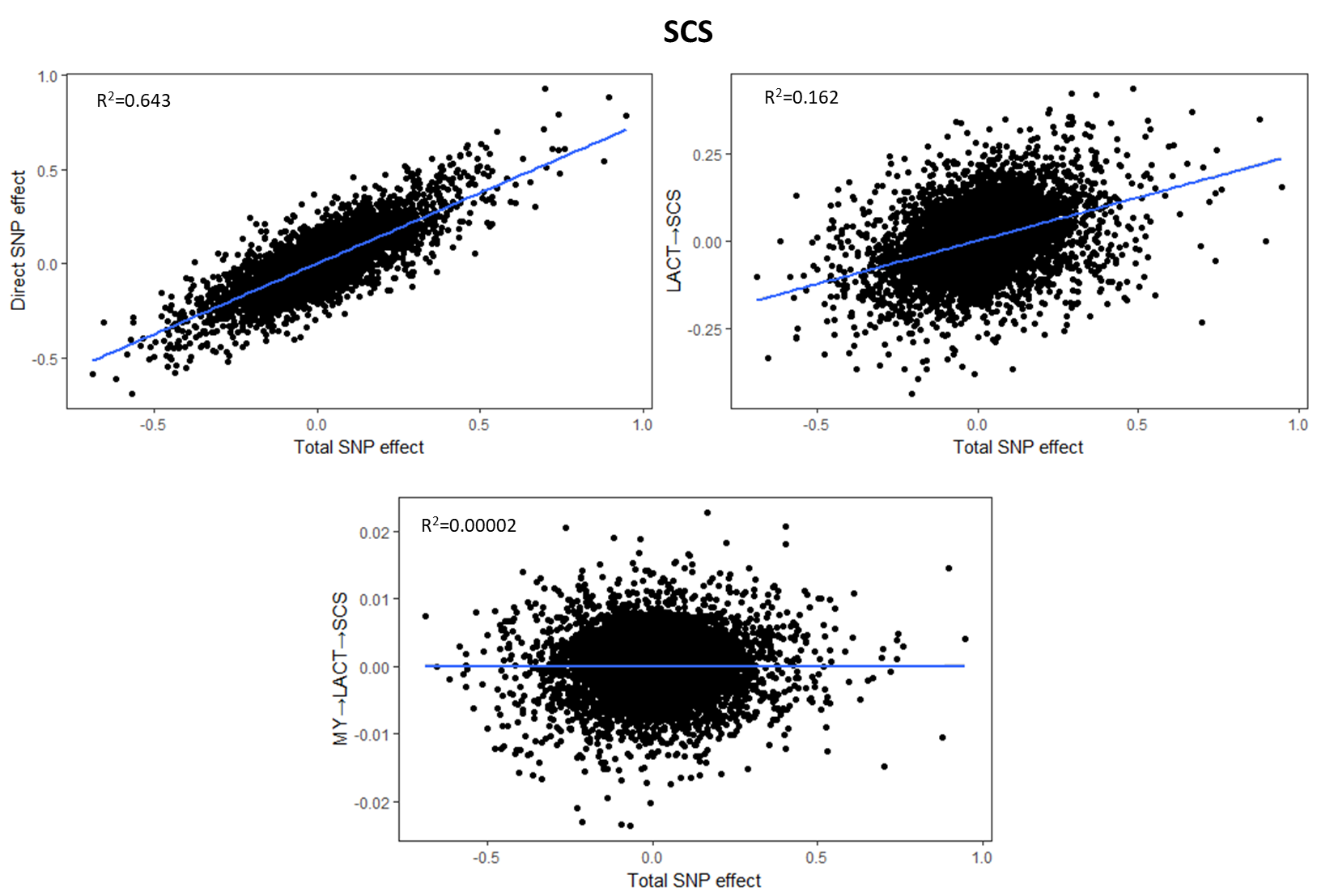


**Supplementary Figure S2.** **Scatter plots of decomposed SNP effects for somatic cell score.** Each point corresponds to the estimated effect of a SNP which affects somatic cell score (SCS) directly or indirectly. LACT: lactose; MY: milk yield.


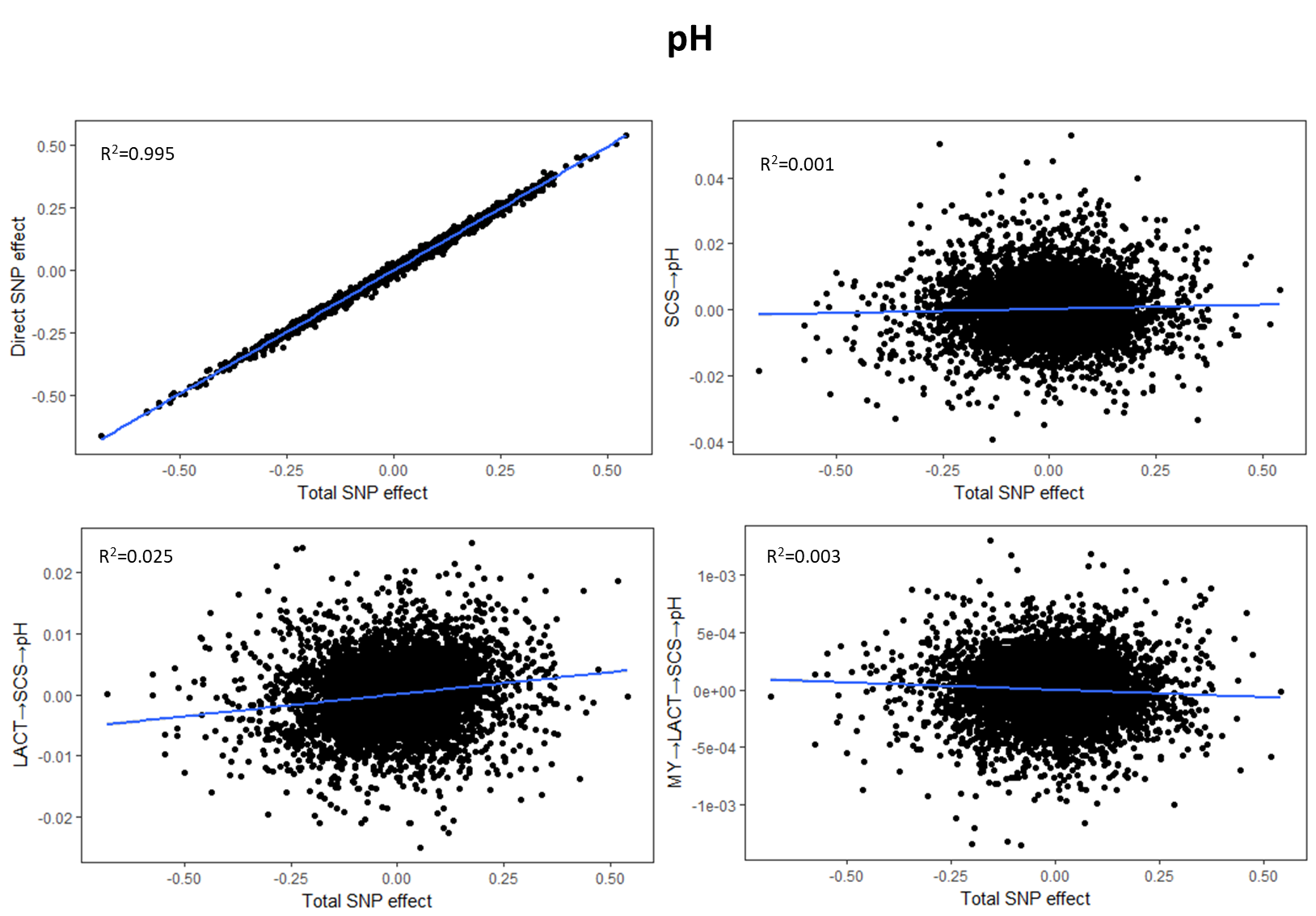


**Supplementary Figure S3.** **Scatter plots of decomposed SNP effects for milk pH.** Each point corresponds to the estimated effect of a SNP which affects milk pH directly or indirectly. SCS: somatic cell score; LACT: lactose; MY: milk yield.


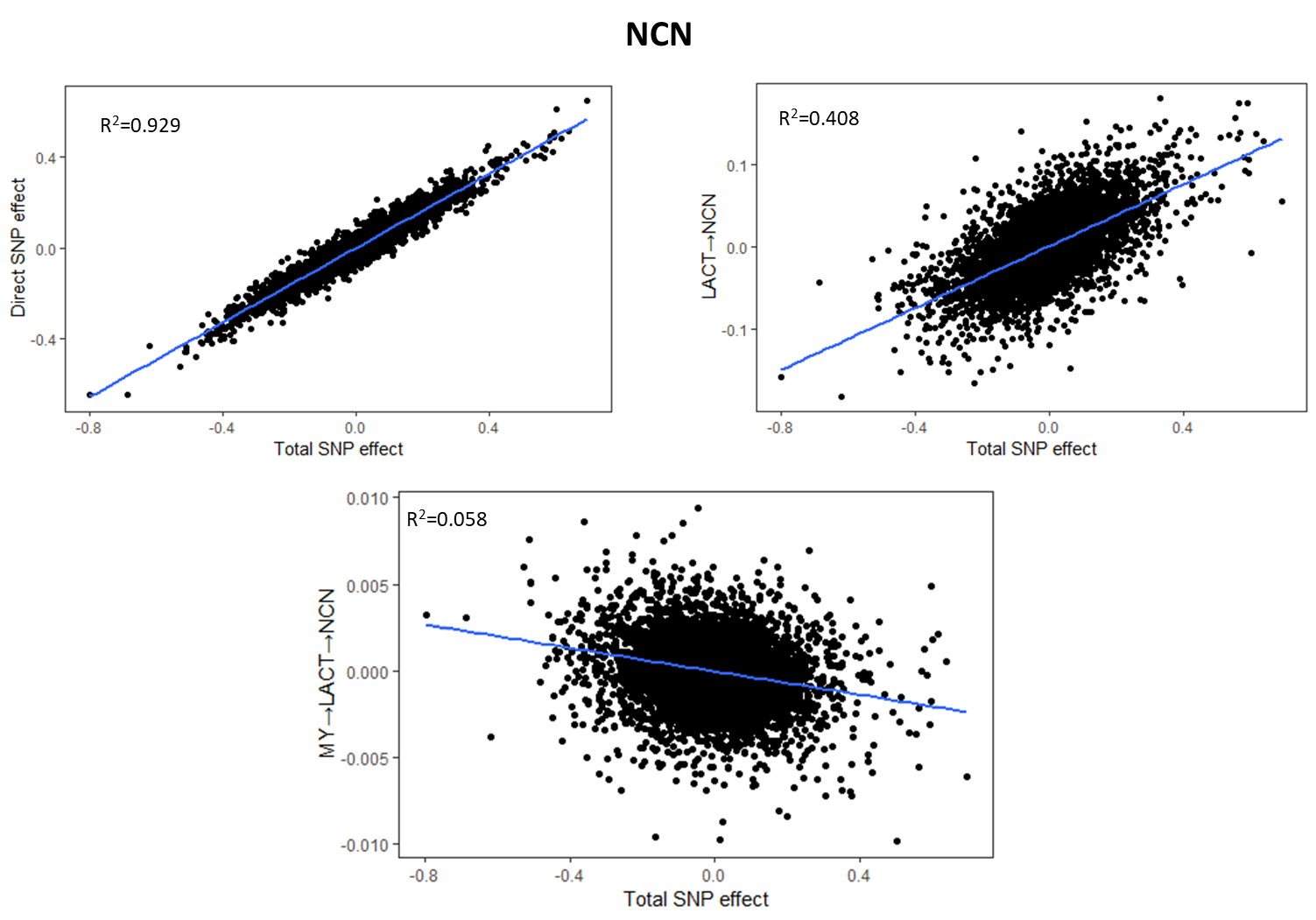


**Supplementary Figure S4.** **Scatter plots of decomposed SNP effects for casein.** Each point corresponds to the estimated effect of a SNP which affects non-casein N (NCN, expressed as % of total milk N) directly or indirectly. LACT: lactose; MY: milk yield.


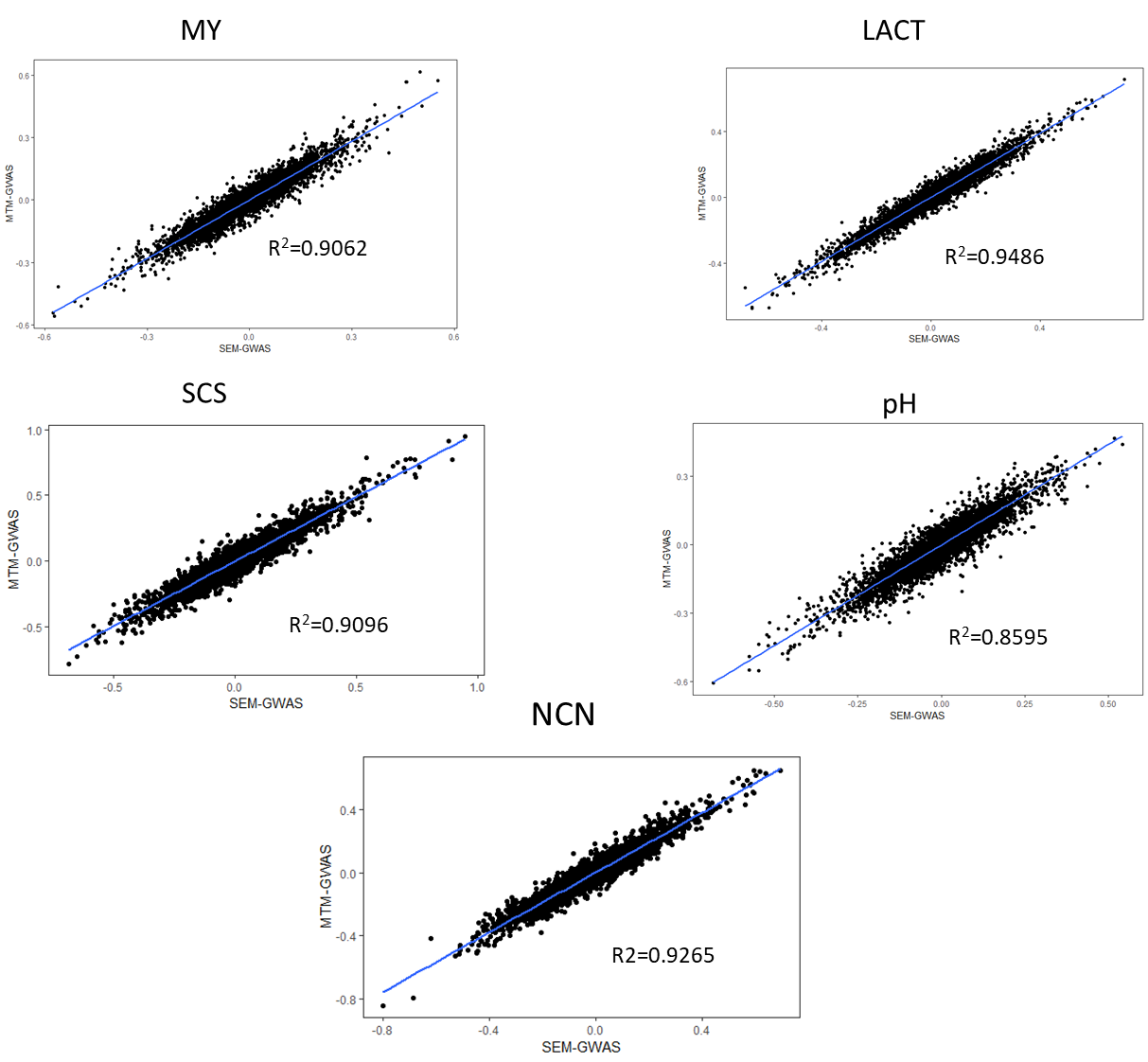


**Supplementary Figure S5.** Comparison of multiple trait (MTM) and fully recursive overall SNP effects obtained from structural equation modeling (SEM)-based GWAS. Overall effects in SEM are the sum of all direct and indirect effects. MY: milk yield; LACT: lactose; SCS: somatic cell score; pH: milk pH; NCN: non- casein N (expressed as % of total milk N).


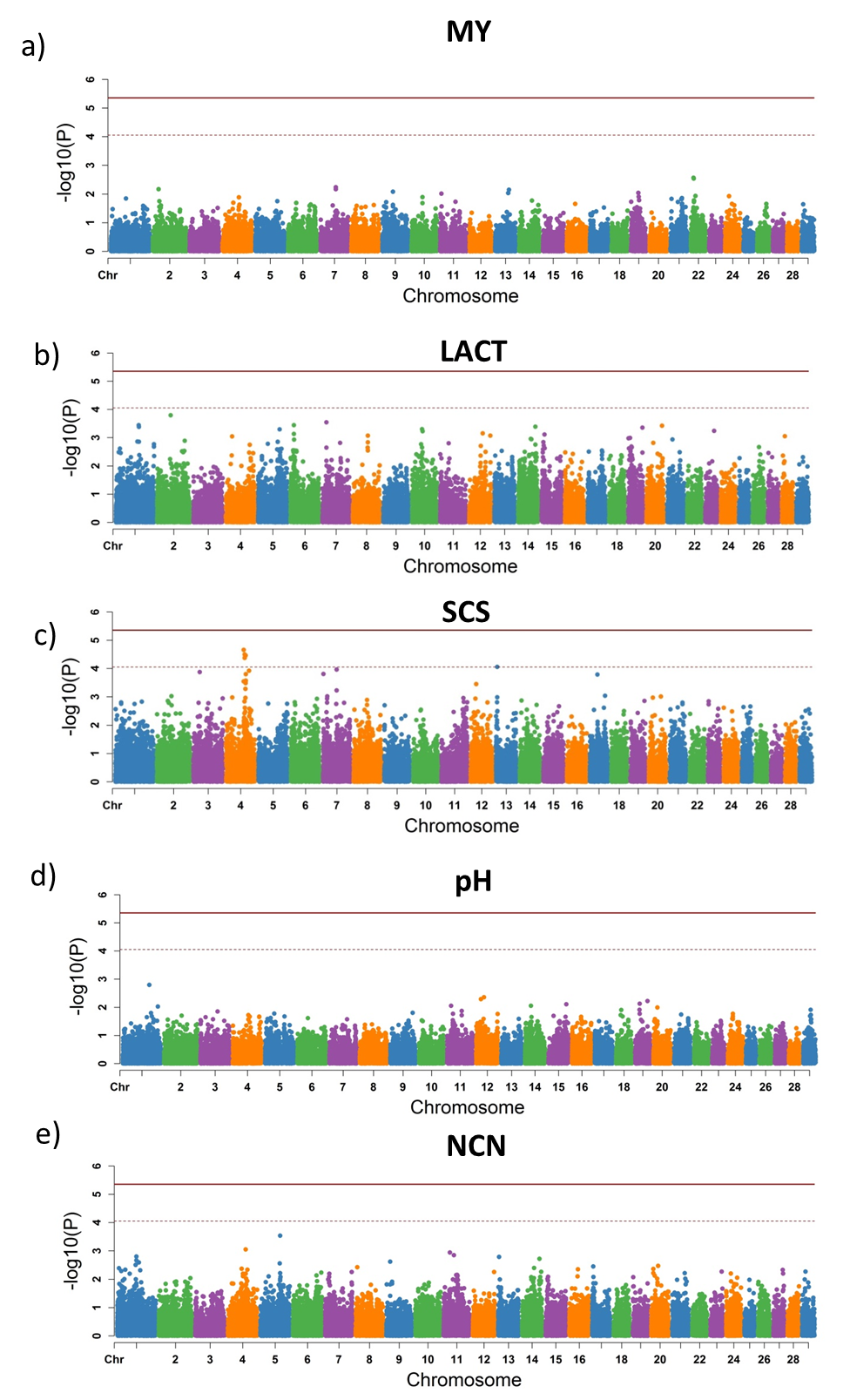


**Supplementary Figure S6. Manhattan plots of significant SNP for the milk related traits using MTM-GWAS.** Each point represents a SNP and the height of the SNP represents the –log10Pvalue for its association with the trait. The continuos line represents the genome-wide significance threshold (5.355) calculated according to the multiple testing adjustment of Li and Ji (2005). The dashed line corresponds to a suggestive significant threshold (4.054). a) milk yield (MY); b) lactose (LACT); c) somatic cell score (SCS); d) pH; e) non-casein N (NCN, expressed as % of total milk N).
